# Temperament and character properties of primary focal hyperhidrosis patients

**DOI:** 10.1186/1477-7525-11-5

**Published:** 2013-01-11

**Authors:** Mehmet Ak, Didem Dincer, Bikem Haciomeroglu, Suleyman Akarsu, Alper Cinar, Nergis Lapsekili

**Affiliations:** 1 Department of Psychiatry, Gulhane School of Medicine, Etlik, Ankara, 06018, Turkey; 2 Department of Dermatology, Gulhane School of Medicine, Ankara, Turkey; 3 Department of Psychology, Gazi University, Ankara, Turkey

**Keywords:** Primary Focal Hyperhidrosis, Temperament, Character, Psychodermatological disorders

## Abstract

**Background:**

Primary focal hyperhidrosis is a health problem, which has negative effects on the patient's quality of life and significantly affects the patients’ daily activities, social and business life. The aim of this study is to evaluate temperament and character properties of patients diagnosed with primary focal hyperhidrosis.

**Methods:**

Fifty-six primary focal hyperhidrosis (22.42 ± 7.80) and 49 control subjects (24.48 ± 5.17) participated in the study. Patients who met the diagnostic criteria for PFH were referred to psychiatry clinic where the subjects were evaluated through Structured Clinical Interview for DSM Disorders-I and Temperament and Character Inventory.

**Results:**

In order to examine the difference between the PFH and control group in terms of temperament and character properties, one-way Multivariate Analysis of Variance (MANOVA) was conducted. In terms of temperament properties, PFH group took significantly higher scores than control group in Fatigability and asthenia dimension. In terms of character properties, PFH group scored significantly lower than control group in Purposefulness , Resourcefulness , Self-Directedness and scored significantly higher than control group in Self-forgetfulness and Self-Transcendence.

**Conclusion:**

Temperament and character features of PFH patients were different from healthy group and it was considered that these features were affected by many factors including genetic, biological, environmental, socio-cultural elements. During the follow-up of PFH cases, psychiatric evaluation is important and interventions, especially psychotherapeutic interventions can increase the chances of success of the dermatological treatments and can have a positive impact on the quality of life and social cohesion of chronic cases.

## Background

Primary focal hyperhidrosis (PFH) is a disorder of excessive, bilateral and relatively symmetric sweating typically localized to specific body areas such as axillae, palms and soles. For the diagnosis of PFH only excessive sweating in those regions is not enough. Sweating must be visible, continuing for at least six months without an underlying disease and have at least two other diagnostic criteria for PFH [1-3]. It is estimated that it affects about 1% or even more of the general population [4]. The true etiology of this disorder remains unknown. PFH may represent a complex dysfunction of the autonomic nervous system, involving both the sympathetic and parasympathetic pathways [5]. Psychiatric symptoms, such as depression, anxiety and stress have frequently been implicated to play a role in the genesis of the disease as well as in its evolution [6]. However, limited number of studies has systematically assessed psychopathology in PFH and has yielded mixed results due to small sample sizes and the utilization of suboptimal measures of personality [4-6].

Cloninger's psychobiological model of personality has recently been widely used to examine the cases with dermatologic diseases. In this model, personality is composed of four temperament and three character dimensions. Genetic structure affects these temperament dimensions by the rate of 40-60% and character dimensions by the rate of 10-15%, while environmental factors affect temperament and character dimensions by the rate of 30-35% [7,8]. In addition, the relationship between temperament dimensions and neurotransmitter systems is emphasized [9]. Therefore, detection of a possible relationship between the structure of temperament and character and psychocutaneous diseases will help in planning the treatment and perhaps the prevention of the disease.

Only one study was found which has investigated the temperament and character properties of PFH patients. The results of the study revealed that the total novelty seeking score in hyperhidrosis was significantly lower than in controls. The total reward dependence and persistence scores were significantly higher in hyperhidrosis patients. Regarding character dimensions, the total score in each of the subscales self-directedness, cooperativeness and self-transcendence was found to be higher in hyperhidrosis patients [5].

Our aim in this study is to evaluate temperament and character properties of patients diagnosed with primary focal hyperhidrosis, and to compare the findings with a healthy control group.

## Method

### Procedure

Seventy-one patients, who applied to the dermatology clinic of Gulhane School of Medicine, Ankara, Turkey, with complaints of focal excessive sweating in their hands, feet or axilla, were evaluated by a dermatologist. In order to eliminate the possible secondary causes such as hyperthyroidism, hiperpitutiarizm, diabetes mellitus, the analysis including of blood count, routine, fasting blood glucose, thyroid hormones, cortisol and PTH were conducted. Patients who met the diagnostic criteria for PFH were referred to psychiatry clinic where the subjects were evaluated through Structured Clinical Interview for DSM Disorders-I (SCID-I) and Temperament and Character Inventory (TCI). Control group consisted of healthy voluntary individuals who had no psychiatric or physical disease. Control group is formed by the individuals accompanying the patients in the hospital from different clinics. Control group also evaluated via SCID-I and TCI.

### Participants

Fifteen out of 71 patients were excluded from the study due to diabetes mellitus (n=2), hyperthyroidism (n=1), hyperpituitarism (n=1), and psychiatric disorders [major depression (n=4), panic disorder (n=3) generalized anxiety disorder (n=2), social anxiety disorder (n= 2)]. Fifty six primary focal hyperhidrosis and 49 control subjects ranging from 17 to 61 years old participated in the study. In terms of age, PFH group (22.42 ± 7.80) was not significantly different from control group (24.48 ± 5.17), t(103)=−1.15, n.s. Socio-demographic characteristics of the sample were presented in Table 1. GATA Ethics Committee approved this study on 05.07.2011.

**Table 1:** Socio-demographic Characteristics of the Sample

	**PFH group (n=56)**	**Control group (n=49)**
Gender		
Male	n=45 (80%)	n=37 (75%)
Female	n=11 (20%)	n=12 (25%)
Age	M=22.42±7.80	M=24.48±5.17
Primary & Secondary School	n=8 (15%)	n=5 (10%)
High School	n=25 (45%)	n=23 (48%)
University and above	n=23 (40%)	n=21 (42%)

### Measurement Tools

*Temperament and Character Inventory (TCI):* Temperament and Character Inventory [10] is a self-assessment tool consists of 240 items rated as “true” or “false”. Temperament has four dimensions, defined as Novelty Seeking (NS), Harm Avoidance (HA), Reward Dependence (RD), and Persistence (P). Character has three dimensions as Self-Directedness (SD), Cooperativeness (C), and Self-Transcendence (ST). Kose et al. [11] and Arkar [12] conducted Turkish reliability and validity study of the scale.

### Statistical Analysis

Statistical analysis was performed using SPSS for Windows 16.0. (SPSS Inc, Chicago, IL). One-way Multivariate Analysis of Variance (MANOVA) was conducted to examine the differences between the PFH and control groups in terms of temperament and character properties.

## Results

In order to examine the difference between the PFH and control group in terms of temperament and character properties, one-way Multivariate Analysis of Variance (MANOVA) was conducted. Results revealed that group (PFH and control) had a main effect on temperament and character properties [Multivariate F(25,67)=1.55, p < .05; Wilks’ Lambda=.63; partial η^2^=.36]. Univariate analyses were examined to find out group differences on temperament and character dimensions with Bonferroni adjustment. Alpha levels lower than 0.0016 were accepted as significant. In terms of temperament properties, PFH group took significantly higher scores than control group in Fatigability and asthenia (HA4). In terms of character properties, PFH group scored significantly lower than control group in Purposefulness (SD2), Resourcefulness (SD3), Self-Directedness (SD), and scored significantly higher than control group in Self-forgetfulness (ST1) and Self-Transcendence (ST) dimensions. Results of the MANOVA were presented in Table 2.

**Table 2:** Comparison of PFH and Control Groups in terms of Temperament and Character Properties

**TCI**	**PFH Group**	**Control Group**	**Univariate F (1, 91)**
	**(Mean± SD)**	**(Mean± SD)**	
Exploratory excitability (NS1)	5.75±0.30	6.27±0.32	1.35
Impulsiveness (NS2)	4.12±0.29	4.06±0.30	0.02
Extravagance (NS3)	4.77±0.26	4.88±0.27	0.09
Disorderliness (NS4)	3.77±0.21	3.72±0.23	0.02
Novelty Seeking (NS)	18.42±0.64	18.95±0.67	0.32
Anticipatory worry (HA1)	5.36±0.31	4.75±0.33	1.77
Fear of uncertainty (HA2)	4.26±0.25	4.36±0.26	0.07
Shyness with strangers (HA3)	3.59±0.32	3.59±0.33	0.01
Fatigability and asthenia (HA4)	4.77±0.30	3.18±0.32	12.84*
Harm Avoidance (HA)	18.00±0.94	15.00±1.00	2.35
Sentimentality (RD1)	7.04±0.26	6.18±0.28	4.85
Attachment (RD3)	4.04±0.26	4.84±0.28	4.19
Dependence (RD4)	2.75±0.20	3.13±0.21	1.62
Reward Dependence (RD)	13.83±0.39	14.15±0.41	0.32
Persistence (P)	4.73±0.24	5.02±0.26	0.64
Responsibility (SD1)	4.30±0.32	5.59±0.33	7.58
Purposefulness (SD2)	4.61±0.24	5.97±0.25	15.31*
Resourcefulness (SD3)	2.87±0.19	3.88±0.20	12.80*
Self-acceptance (SD4)	5.51±0.35	6.61±0.37	4.58
Congruent second nature (SD5)	8.44±0.30	9.09±0.32	2.07
Self-Directedness (SD)	25.75±1.06	31.15±1.12	12.23*
Acceptance (C1)	5.67±0.23	6.61±0.25	7.36
Empathy (C2)	4.36±0.22	4.61±0.23	0.56
Helpfulness (C3)	4.87±0.19	5.15±0.20	1.02
Compassion vs. revenge (C4)	6.89±0.37	7.36±0.39	0.74
Intergraded conscience (C5)	6.51±0.21	6.84±0.22	1.16
Cooperativeness (C)	28.32±0.89	30.59±0.94	3.05
Self-forgetfulness (ST1)	6.42±0.35	4.59±0.37	12.66*
Transpersonal identification (ST2)	5.10±0.32	3.77±0.33	8.10
Spiritual acceptance (ST3)	7.10±0.41	5.63±0.43	5.89
Self-Transcendence (ST)	18.63±0.89	14.00±0.94	12.66*

*p < 0.0016.

## Discussion

Limited number of research related to psychopathology in hyperhidrosis gave conflicting results. Although some studies found that patients with hyperhidrosis have no psychopathology and symptoms of anxiety, depression, and social isolation [4], other studies showed that hyperhidrosis is common in social anxiety disorder, and patients with hyperhidrosis had more disability, fear, avoidance, and other arousal symptoms [13]. The current study aimed to examine the temperament and character properties that might be related to hyperhidrosis.

Temperament refers to automatic emotions and responses thought to be moderately heritable, therefore largely genetically based [14]. Among the temperament properties, Harm Avoidance (HA) reflects the effectiveness of the behavioral inhibition system mediated by serotonin. This temperamental trait is related to the individual’s ability to cope with potentially harmful life events [8]. In terms of fatigability and asthenia (HA4), which is one of the HA dimensions, patients with PFH scored significantly higher than control group, indicating PFH patients might express less energy and have a tendency to tiredness and recover slowly from stressors and illnesses [10].

Character is largely environmentally derived and responsive to learning and maturation. Among the character dimensions, Self-directedness (SD) measures differences in individual’s executive functions. Highly self-directed persons are described as mature, strong, self-sufficient, responsible, reliable, goal-oriented, constructive, and well-integrated individuals when they have the opportunity for personal leadership. They have good self-esteem and self-reliance. On the other hand, individuals who are low in self-directedness are described as immature, weak, fragile, blaming, destructive, ineffective, irresponsible, unreliable, and poorly integrated when they are not conforming to the direction of a mature leader. They are frequently decsribed by clinicians as immature or having a personality disorder [8,10]. Lower scores in Purposefulness (SD2), Resourcefulness (SD3), and total Self Directedness (SD) scores indicated that PFH patients might have difficulty to define, set, and pursue meaningful goals [8,10]. Low self-directedness scores of PFH cases seem compatible with results of other similar psychosomatic diseases [15-17]. It is inevitable that these individual’s success to tolerate stress and to cope with stress is low. This feature can be a reflection of a biological string which is similar to PFH and can be proven with spatial studies including brain imaging. Moreover, PFH patients scored significantly higher than controls in Self-forgetfulness (ST1) and total Self Transcendence (ST). Individuals who have high scores on these subscales are tend to transcend their self-boundaries when deeply involved in a relationship or when concentrating on what they are doing, they are usually described as creative and original, have experience and extraordinarily strong connection to nature and the universe as a whole, and spiritually oriented [8,10]. High self-transcendent individuals are generally more fair, considerate, religious, and decent. This property is adaptively advantageous for people who face death or illnesses, which are inevitable with age [11,18]. Contrary to the temperament properties, which might be related to comorbid psychiatric symptoms, the balance might be kept with this protective and adaptive character property.

Although it is considered that emotional factors have obvious role in the formation, maintenance and exacerbation of psychosomatic skin diseases, personality profile that shows the basic building blocks of these cases, has not been studied enough. To our knowledge, there is one study on this issue in the patients diagnosed with PFH [5]. In Karaca et al.’s study, the results showed that the total Novelty Seeking score in hyperhidrosis patients was significantly lower than in controls. There was no significant difference in total Harm Avoidance scores between hyperhidrosis patients and controls, except for the Fear of Uncertainty which was found to be significantly greater in hyperhidrosis patients. The total Reward Dependence and Persistence scores were significantly higher in hyperhidrosis patients [5]. In terms of character properties, Karaca et al. found that the total score in each of the subscales Self-directedness, Cooperativeness and Self-transcendence was found to be higher in hyperhidrosis patients [5]. Similarly, in the current study hyperhidrosis patients exhibited higher Self-transcendence scores than control group, however, contrary to their findings Self- directedness scores were found to be lower in hyperhidrosis patients compared to control group. These conflicting findings might be due to the age and number of participants, and the statistical procedure that was used.

The review of studies on other psychosomatic skin diseases, which used Temperament and Character Inventory (TCI) revealed contradictory results. Kılıç et al. found higher Harm Avoidance scores and lower Self-directedness scores in patients with psoriasis than healthy controls [15]. In contrast, Güler et al. found that temperament properties of psoriasis, vitiligo, and neurodermatitis cases did not differ from each other and their character scores were similar to the healthy controls [19]. Ak et al. reported significantly higher Novelty Seeking, Harm Avoidance, Reward Dependence, and Self-transcendence scores in male psoriasis patients compared to control group [20].

Although this study excluded the patients with a psychiatric diagnosis (since it was a requirement for the diagnosis of PFH), it is a significant deficiency of the study that the level of depression and anxiety symptoms of the patients was not measured and so that the relationship between these symptoms and temperament-character properties was not evaluated. Moreover, since it is a cross-sectional study, the findings are not sufficient to establish a cause and effect relationship.

To our knowledge, this study is the second one investigating the temperament-character properties in patients with PFH. As a result of the study, it was seen that temperament and character features of PFH patients were different from healthy group and it was considered that these features were affected by many factors including genetic, biological, environmental, socio-cultural elements. During the process of understanding the **pathophysiology of PFH, all emphasis is on “abnormal or exaggerated central response to normal emotional** stress”. Mental reflection of the biological mechanism underlying this might be the personality traits. During the follow-up of these cases, psychiatric evaluation is important and interventions in these areas are required. It is considered that especially psychotherapeutic interventions can increase the chances of success of the dermatological treatments and can have a positive impact on the quality of life and social cohesion of chronic cases. In the future, longitudinal studies containing biological assessments (genetic, brain imaging) will provide different and valuable data.

## Competing interests

The authors declare that they have no competing interests.

## Authors’ contributions

MA have made substantial contributions to conception and design, DD contributed to the acquisition of data, BH conducted the statistical analysis and interpretation of data, and drafting the manuscript and revising, SA, AC and NL contributed to the acquisition of data, and drafting the manuscript. All authors read and approved the final manuscript.
